# Parturient Apoplexy*Read before the California State Veterinary Association.

**Published:** 1894-09

**Authors:** F. E. Pierce

**Affiliations:** Oakland, Cal.


### PARTURIENT APOPLEXY.*

By F. E. Pierce, D.V.S.

While nearly all of the papers that have been read at our meetings of late have been on some contagious or infectious disease, I thought I would take up a more common subject, one we all meet with very often in our practice, and one which gives us as much trouble as almost any other disease we meet.

The disease which I shall present to you for consideration is Parturient Apoplexy, or Milk Fever, the term usually applied to it by dairymen. It is a malady usually attended with fatal results, and is regarded with more dread by cattle owners than any other disease which the cow is subject to. Some herds of cattle are more subject to it than others. The majority of cases coming under my notice have been among the high bred Jerseys.

It is not my purpose to advance any new theory or treatment of this disease, but simply to bring out the views of the different members through the discussion which I hope will follow.

Causes: The causes are predisposing and exciting. Among the first may be mentioned high condition; well fed cows, and especially large milkers, suffer from this disease, and one attack usually predisposes another. The exciting cause is the act of parturition; and from my observation I have noticed the disease to follow a case of parturition where there has been very slight labor and the animal being in a plethoric condition. And in my experience in getting the history, the owner says, almost always, that the cow had a very easy time calving, and cleaned in a very short time; and also that the appetite was good previous to calving, and for twenty-four to forty-eight hours following; while on the contrary, I cannot personally call to mind one case of parturient apoplexy following a long and protracted case of parturition.

The pathology of parturient apoplexy appears to be largely a matter of conjecture, even with our leading writers on scientific subjects. Some animals, having a mild attack, recover with ordinary intelligent treatment, while others, having it in a more severe form, succumb in spite of all our efforts.

*Read before the California State Veterinary Association.

It is a parturient disease, characterized by a sudden suppression of milk, congestion of the brain and apoplexy.

The excess of blood in the system which, before calving, went to supply the fœtus, instead of being diverted to the milk producing channels, has been thrown back upon the system, and has a determination to the brain. This produces a state of coma, which has to run its course, and which terminates either favorably or unfavorably.

Symptoms: –The first symptom usually occurs in from one to three days after calving. There will be a very sudden diminished secretion of milk, loss of appetite, and rumination ceases.

The respiration becomes hurried and plaintive. There will be knuckling at the fetlock, staggering gait, sways from side to side, and finally falls, very frequently never to rise again.

Treatment:—Many remedies have been prescribed. Some are good, others are worse than useless. Order the animal well bedded, and have sacks of straw packed around her, in order to keep her well upon the sternum. If called early administer magnesia sulph. one lb. Give enemas of warm water and soap, and if, upon examination, you find that the urine is not passed freely, it should be drawn with the catheter, at intervals not exceeding six hours apart. If deglutition is not impaired, give stimulants at frequent intervals. If you do not get any action from your purgative, I recommend giving sulph. eserine gr. 1, and repeat in gr. ss doses every hour, until 2½ grs. have been given. Apply mustard to the spinal column, and cold water or cracked ice to the head. As soon as the cow is able to rise, and her bowels have resumed their functions, give light, sloppy food for a few days, and follow up for several days with tonics.

Preventive Measures:—Give a mild saline laxative, from three to five days previous to calving, and in many instances all trouble may be avoided, thus verifying the old saying, that "an ounce of prevention is worth a pound of cure."

OAKLAND, Cal.

